# Different Gut Microbial Profiles in Sub-Saharan African and South Asian Women of Childbearing Age Are Primarily Associated With Dietary Intakes

**DOI:** 10.3389/fmicb.2019.01848

**Published:** 2019-08-14

**Authors:** Minghua Tang, Daniel N. Frank, Antoinette Tshefu, Adrien Lokangaka, Shivaprasad S. Goudar, Sangappa M. Dhaded, Manjunath S. Somannavar, Audrey E. Hendricks, Diana Ir, Charles E. Robertson, Jennifer F. Kemp, Rebecca L. Lander, Jamie E. Westcott, K. Michael Hambidge, Nancy F. Krebs

**Affiliations:** ^1^Section of Nutrition, Department of Pediatrics, University of Colorado Denver, Aurora, CO, United States; ^2^Division of Infectious Diseases, School of Medicine, University of Colorado Denver, Aurora, CO, United States; ^3^Kinshasa School of Public Health, Kinshasa, Democratic Republic of the Congo; ^4^KLE Academy of Higher Education and Research’s Jawaharlal Nehru Medical College, Belagavi, India; ^5^Department of Mathematical and Statistical Sciences, University of Colorado Denver, Aurora, CO, United States

**Keywords:** microbiota, Democratic Republic of the Congo, India, diet, Women

## Abstract

**Background:**

To compare and characterize the gut microbiota in women of childbearing age from sub-Saharan Africa (the Democratic Republic of the Congo, DRC) and South Asia (India), in relation to dietary intakes.

**Methods:**

Women of childbearing age were recruited from rural DRC and India as part of the Women First (WF) preconception maternal nutrition trial. Findings presented include fecal 16S rRNA gene-based profiling of women in the WF trial from samples obtained at the time of randomization, prior to initiation of nutrition intervention and to conception.

**Results:**

Stool samples were collected from 217 women (DRC *n* = 117; India *n* = 100). Alpha diversity of the gut microbiota was higher in DRC than in India (Chao1: 91 ± 11 vs. 82 ± 12, *P* = 6.58E-07). The gut microbial community structure was not significantly affected by any demographical or environmental variables, such as maternal BMI, education, and water source. *Prevotella, Succinivibrio*, and *Roseburia* were at relatively high abundance without differences between sites. *Bifidobacterium* was higher in India (4.95 ± 1.0%) than DRC (0.3 ± 0.1%; *P* = 2.71E-27), as was *Lactobacillus* (DRC: 0.2 ± 0.0%; India: 1.2 ± 0.1%; *P* = 2.39E-13) and *Faecalibacterium* (DRC: 6.0 ± 1.7%; India: 8.4 ± 2.9%; *P* = 6.51E-7). *Ruminococcus* was higher in DRC (2.3 ± 0.7%) than in India (1.8 ± 0.4%; *P* = 3.24E-5) and was positively associated with consumption of flesh foods. *Succinivibrio* was positively associated with dairy intake in India and fish/insects in DRC. *Faecalibacterium* was positively associated with vitamin A-rich fruits and vegetables. Overall, these observations were consistent with India being primarily vegetarian with regular fermented dairy consumption and DRC regularly consuming animal-flesh foods.

**Conclusion:**

Consumption of animal-flesh foods and fermented dairy foods were independently associated with the gut microbiota while demographic variables were not, suggesting that diet may have a stronger association with microbiota than demographic characteristics.

## Introduction

The gut microbiota plays a critical role in human health and disease (Turnbaugh et al., 2006, 2009), and can be impacted by multiple factors, such as diet, socioeconomic status, ethnicity, and geographical locations (Gupta et al., 2017). Among these variables, diet and geographical locations usually overlap with each other and appear to be the most influential determinants of the gut microbiota (Rothschild et al., 2018). For example, compared with African Americans who consume a western diet, native Africans have higher fiber and lower protein intakes, which are associated with higher abundances of *Prevotella* and lower abundance of *Bacteroides*, and also a lower risk of colon cancer (O’Keefe et al., 2007; Ou et al., 2013). These different microbial profiles have been reported in other studies comparing populations in two or more distinctive geographical locations such as Africa vs. Europe/United States. One cohort study showed significant variations in gut microbiota of Malawians and Amerindians, compared with non-Amerindian United States adults. For instance, non-Amerindian United States adults were characterized by less *Prevotella* and greater *Bacteroides* as well as reduced diversity compared with the Malawians and Amerindians (Yatsunenko et al., 2012). Geography-specific features could interact with other influencing factors of the gut microbiota. For example, European, but not South Asian, subjects with inflammatory bowel disease exhibit increased abundances of Firmicutes (Rehman et al., 2016). Even within Southeast Asia, infants in Singapore and Indonesia have different microbial community structures (Yap et al., 2011). Overall, these findings suggest that geographical locations may have an independent effect on modulating the structure of the gut microbiota, which may be associated with health status (Gupta et al., 2017) and thus needs to be carefully characterized.

Most of the current literature characterizing the gut microbiota has studied urban/westernized settings, or compared geographical locations as rural/agricultural vs. urban/westernized settings (O’Keefe et al., 2007; Yatsunenko et al., 2012; Ou et al., 2013). Limited research has focused on rural/developing countries, especially comparing two developing regions of contrasting diets and locations. The Women First (WF) Preconception Nutrition Trial recruited women of reproductive age living in under-resourced environments to participate in a preconception lipid-based micronutrient supplementation trial (Hambidge et al., 2014, 2019). The objective of this study was to characterize and compare the gut microbiotas in participants at baseline from two sites of the WF trial, Africa (the Democratic Republic of the Congo or DRC) and South Asia (India), in relation to dietary intakes (24-h recall questionnaires), demographic, and hygiene factors. We hypothesized that both the diversity and composition of gut microbiotas would differ between DRC and India, and that the differences would be associated with dietary intakes.

## Materials and Methods

### Participants

Women of childbearing age were recruited from rural DRC (Equateur Province) and India (Belagavi, Karnataka) as part of the WF preconception maternal nutrition trial (Hambidge et al., 2014, 2019). Inclusion criteria were 16–35 years of age; parity 0–5; expectation to have first or additional pregnancy within next 2 years and without intent to utilize contraception. Enrollment occurred after screening, and informed consent was obtained by the home visitor research assistant if the potential participant was eligible. Findings presented in the current report include the gut microbiota of women at the time of randomization prior to the initiation of the nutrition intervention and at least 3 months prior to conception in the WF trial and represented participants from two of the WF sites, with distinctive ethnicity, diet, culture and geographical locations. Women were recruited from 12 villages in rural DRC and 9 villages from rural India. The project was approved by the Colorado Multiple Institutional Review Board, University of Colorado, the local and/or national ethics committees for each site (registered with the US Office of Human Research Protection and with Federal-wide Assurance in place). Written informed consent was obtained from all participants and the study was registered at ClinicalTrials.gov (NCT01883193).

Questionnaires of demographic information were administered: cell phone (Yes/No), education (None vs. At least secondary), electricity (Yes/No), man-made floor (Yes/No), flush toilet (Yes/No), improved water (Yes/No), landline (Yes/No), motorcycle (Yes/No), fridge (Yes/No), worry no food (Yes/No), and open sewage near house (Yes/No). Improved water means the participant had access to filtered or treated water. Questionnaires were administered in the home by the local home visitor research assistant and were completed within 1 week of enrollment. A mobile assessment team obtained past medical history and height and weight, from which body mass index (BMI) was calculated.

### Stool Sample Collection

A stool sample was collected from each participant (DRC *n* = 117; India *n* = 100). A pre-labeled fecal bag, Ziploc bag, a black cryogenic pen, and a Styrofoam storage box containing ice or ice packs were provided to each participant. Stool was collected into the fecal bag using a sterile scoop and then placed into a second Ziploc bag. Participants then placed the bag into the Styrofoam storage container until picked up by the research team on the day the stool was passed. When receiving samples, the research team labeled the sample date and time of stool passage. The research team scooped about a teaspoon of stool and transferred the sample to a sterile stool storage tube with 3 ml RNAlater^TM^ (ThermoFisher Scientific Inc., Waltham, MA, United States), ensuring that the specimen was coated with RNAlater^TM^ and that the label was complete. The stool samples were then frozen at −20°C or colder. Samples were shipped to the University of Colorado on ice packs or at ambient temperature.

### High-Throughput DNA Sequencing for Microbiome Analysis

#### 16S Amplicon Library Construction

Bacterial profiles were determined by broad-range amplification and sequence analysis of 16S rRNA genes following our previously described methods (Hara et al., 2012; Markle et al., 2013). In brief, DNA was extracted from 25 to 50 mg of stool using the QIAamp PowerFecal DNA kit (Qiagen Inc., Carlsbad, CA, United States), which employs chemical and mechanical disruption of biomass. PCR amplicons were generated using barcoded (Frank, 2009) primers that target approximately 450 basepairs of the V3V4 variable region of the 16S rRNA gene (338F: 5′ACTCCTACGGGAGGCAGCAG and 806R: 5′ GGACTACHVGGGTWTCTAAT) (Lane et al., 1985; Weisburg et al., 1991). PCR products were normalized using a SequalPrep^TM^ kit (Invitrogen, Carlsbad, CA, United States) and then pooled. The amplicon pool was partially lyophilized to reduce its volume then purified and concentrated using a DNA Clean and Concentrator Kit (Zymo, Irvine, CA, United States). Pooled amplicons was quantified using a Qubit Fluorometer 2.0 (Invitrogen, Carlsbad, CA, United States). Illumina paired-end sequencing was performed following the manufacturer’s protocol on the MiSeq platform using a 600 cycle version 3 reagent kit and versions v2.4 of the MiSeq Control Software.

#### Analysis of Illumina Paired-End Reads

Illumina Miseq paired-end reads were aligned to human reference genome hg19 with bowtie2 and matching sequences discarded (Homo Sapiens Ucsc Hg19 Human Genome Sequence from iGenome: Illumina, 2009; Langmead and Salzberg, 2012). As previously described, the remaining non-human paired-end sequences were sorted by sample via barcodes in the paired reads with a python script (Markle et al., 2013). The sorted paired reads were assembled using phrap (Ewing and Green, 1998; Ewing et al., 1998). Pairs that did not assemble were discarded. Assembled sequence ends were trimmed over a moving window of five nucleotides until average quality met or exceeded 20. Trimmed sequences with more than 1 ambiguity or shorter than 350 nt were discarded. Potential chimeras identified with Uchime (usearch6.0.203_i86linux32) (Edgar et al., 2011) using the Schloss (Schloss and Westcott, 2011) Silva reference sequences were removed from subsequent analyses. Assembled sequences were aligned and classified with SINA (1.3.0-r23838) (Pruesse et al., 2012) using the 418,497 bacterial sequences in Silva 115NR99 (Quast et al., 2013) as reference configured to yield the Silva taxonomy. Operational taxonomic units (OTUs) were produced by clustering sequences with identical taxonomic assignments. The software package Explicet (v2.10.5) (Robertson et al., 2013) was used for microbial diversity analysis.

### 24-h Dietary Recall

For those participants who conceived after at least 3 months after randomization and entered the pregnancy phase of the trial, repeat 24-h dietary recalls were conducted in first trimester on a randomly selected subgroup of the study participants. For the current analysis, dietary data were obtained for 50 women at each site (DRC *n* = 50; India *n* = 50). In brief, two 24-h dietary recalls (Lander et al., 2017) were conducted 2–4 weeks apart once pregnancy was confirmed prior to 12-week gestation. The analysis reflected only the participants’ food intakes and did not include any contribution from the trial intervention nutrient supplement. No counseling regarding diet choices or quality was provided over the course of the trial. Dietary assessment training was provided for each of the site nutritionists by the lead study nutritionist. A unique food nutrient composition database was constructed at each site based on the food intake data collected from the dietary recalls to quantify intakes as nutrients and food groups (Lander et al., 2017).

### Statistical Approach

Values are presented as mean ± SD for continuous variables. Alpha diversity indices measured for the gut microbiota were tested using the Mann–Whitney statistic. Beta diversity was calculated using weighted UniFrac distances. To compare between DRC and India for differences of the gut microbial profiles, non-parametric Mann–Whitney tests were used. *P* < 0.05 was considered significant between DRC and India for the gut microbial profiles comparison. Within each site (e.g., DRC and India), permutation-based multivariate analysis of variance (PERMANOVA) tests were used to assess the associations between gut microbial community composition and demographical variables. Associations between the relative abundances of bacterial taxa and nutrients/food-groups were assessed by Spearman rank-order correlation tests. Results were visualized by plotting heatmaps of Spearman’s rho correlation coefficient using the heatmap.2 R function; hierarchical clustering and dendrograms were generated using the default parameters of heatmap.2/dist/hclust functions, using Euclidean distances. Nominal *p*-values not accounting for multiple testing are reported. Data were analyzed using R version 2.7.2 (R Foundation for Statistical Computing; Vienna, Austria).

## Results

### Participants

Recruitment in India started in January 2014 and enrollment was completed in August 2014. For DRC, the first participant was enrolled in April 2014 and enrollment was completed in September 2014. A total of 3810 women were screened and 3553 were enrolled in the WF trial from DRC (*n* = 1732) and India (*n* = 1821). Stool samples were collected at baseline from a random sample of *n* = 217 women (DRC *n* = 117; India *n* = 100). Baseline characteristics are summarized in Table 1. Women in DRC were all African ancestry and women in India were South Asian ancestry (predominantly following Hindu religion). All the demographical variables listed in Table 1 differed between DRC and India, except for BMI. Among these participants, *n* = 50 from DRC and *n* = 50 from India became pregnant and had dietary intake data during first trimester.

**TABLE 1:** Participant characteristics^1^.

	**DRC (*n* = 117)**	**India (*n* = 100)**	***P* value**
Age^2^	23 ± 5 years	22 ± 3 years	*P* = 0.02
BMI^2^	20.5 ± 2.3	19.8 ± 3.7	*P* = 0.08
No education^3^	*n* = 27	*n* = 7	*P* = 0.001
Has electricity^3^	*n* = 0	*n* = 94	*P* < 0.0001
Has cell phone^3^	*n* = 26	*n* = 96	*P* < 0.0001
Has improved water^3^	*n* = 51	*n* = 97	*P* < 0.0001
Has flush toilet^3^	*n* = 0	*n* = 18	*P* < 0.0001
Has open sewage^3^	*n* = 0	*n* = 46	*P* < 0.0001

*^1^Mean ± SD; ^2^Independent samples *t*-test; ^3^Chi-square test.
*

### Dietary Intakes

The main staple of the DRC women was a fermented yellow maize porridge (i.e., “stiff” and “soft” types), often complemented with caterpillars, termites, small fish, and/or peanuts in sparse amounts. Also prominent was the consumption of cassava, both its leaves and flour made from its root, with the addition of palm oil to most recipes. Fruit and vegetables were also significant elements of the diet. Rice was the main staple in India, with frequent consumption of jowar (sorghum) flour roti. In this primarily lacto-vegetarian population, a large variety of legumes were consumed in addition to many types of vegetable “bhaji,” whereas very few animal-flesh foods were eaten. The diets in India included multiple spices (e.g., curry leaves, cumin seeds, red chili peppers, etc.). Buffalo milk tea and yogurt curds were regular features of the diet in Indian women; dairy products were not in the diets of the participants from DRC.

Table 2 summarizes the energy, macronutrient intakes and food groups of these participants at the first trimester based on repeat quantitative 24-h dietary recall (DRC *n* = 50; India *n* = 50). Although BMI was similar between participants from DRC and India, women from India had a lower average body weight than women from DRC at both baseline (44 ± 7 vs. 49 ± 8 kg, *P* = 0.001) and first trimester (45 ± 7 vs. 50 ± 8 kg, *P* = 0.001). Due to the nature of different dietary intakes, DRC and India had different food groups. In DRC, the four most common food groups based on intakes (g/day) were starchy/staples (especially maize and cassava) (1085 ± 570 g/day), vitamin A-rich fruits/vegetables (822 ± 542 g/day), legumes/ground nuts (143 ± 202 g/day), and fish/insects (122 ± 225 g/day). In India, the four most common food groups were staples (especially rice and wheat) (1025 ± 376 g/day), non-fermented dairy (512 ± 266 g/day), pulses (260 ± 164 g/day), and non-vitamin A rich vegetables (117 ± 110 g/day) (Lander et al., 2019).

**TABLE 2:** Energy and nutrient intakes in DRC and India^1^.

	**DRC (*n* = 50)**	**India (*n* = 50)**	***P* value**
Energy (kcal/day)^2^	1983 ± 745	1314 ± 364	*P* < 0.0001
Energy (kcal/kg/day)^2^	41 ± 16	30 ± 10	*P* = 0.0001
Protein (g/day)^2^	41 ± 17	33 ± 12	*P* = 0.01
Protein (g/kg/day)^2^	0.84 ± 0.34	0.75 ± 0.30	*P* = 0.17
Total fat (g/day)	96 ± 51	53 ± 17	*P* < 0.0001
Carbohydrate (g/day)	247 ± 86	181 ± 52	*P* < 0.0001
Total fiber (g/day)	23 ± 9	16 ± 2	*P* < 0.0001

*^1^Mean ± SD; ^2^Indpendent samples *t*-test.
*

### Gut Microbiota Diversity and Richness

**FIGURE 1:**
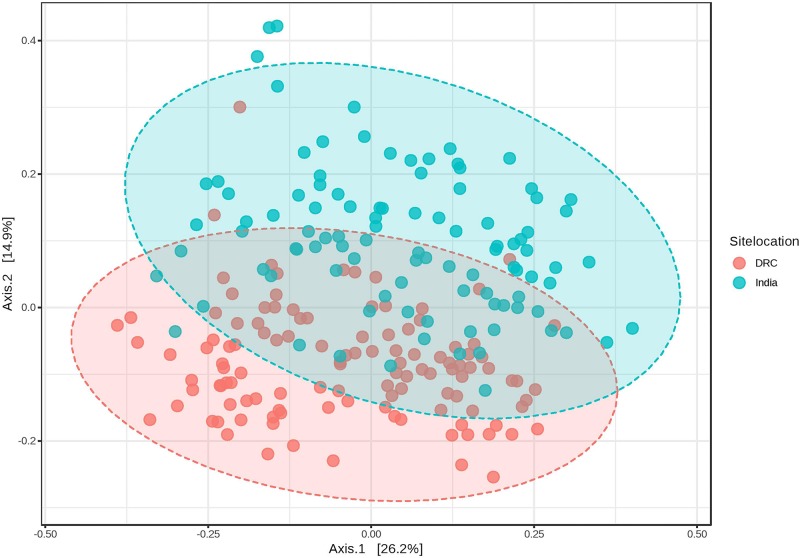
Weighted UniFrac PCoA plots at genus level showing beta diversity of DRC and Indian participants. PERMANOVA *F*-value: 26.439; *R*-squared: 0.10951; *p*-value < 0.001.

The average Good’s index of each sequence library was 99.87 and 99.88% for DRC and India, respectively, indicating the most biodiversity was captured in each library (participant) and the depth of sequencing was sufficient to represent the biodiversity in the specimens. Alpha diversity of the gut microbiota was higher in DRC than in India using the Chao1 richness index (91 ± 11 vs. 82 ± 12, *P* = 6.58E-07) and Shannon diversity index (3.9 ± 0.5 vs. 3.7 ± 0.6, *P* = 0.003). Beta diversity by weighted UniFrac at genus level showed that the gut communities across DRC women were more similar than among Indian women (*P* < 0.001). Figure 1 shows the dissimilarity matrix by Principal Coordinate Analysis.

### Gut Microbiota Community Structure

**FIGURE 2:**
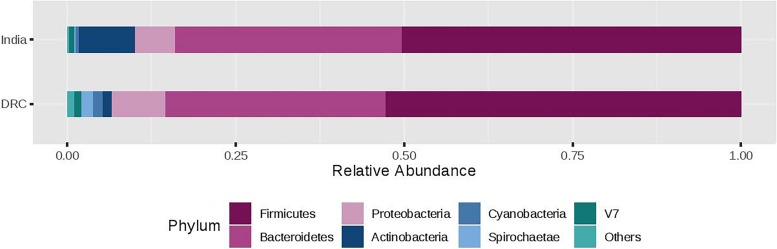
Relative abundance of the gut microbiota phylum between India and DRC. Stacked bar represented percentage abundance. Small taxa with counts less than 150000 were merged as others.

**FIGURE 3:**
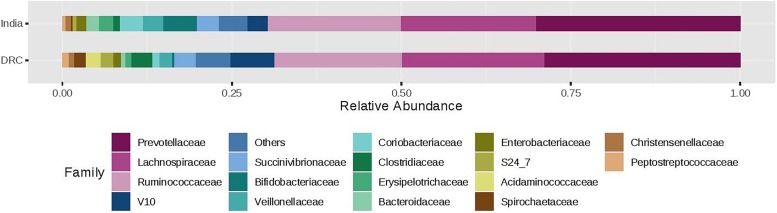
Relative abundance of the gut microbiota families between India and DRC. Stacked bar represented percentage abundance. Small taxa with counts less than 150000 were merged as others.

The three most abundant phyla were Firmicutes, Bacteroidetes, and Proteobacteria with average relative abundances at 51, 33, and 7%, respectively and there was no statistical difference between DRC and India (Figure 2). These three phyla accounted for over 90% of the overall bacterial abundance. The next most abundant phylum was Actinobacteria, which exhibited elevated relative abundance in India (8.3 ± 2.5%) compared to DRC (1.4 ± 0.8%, *P* = 1.29E-27). At the family level (Figure 3), the three most abundant taxa were *Prevotellaceae* (DRC: 29 ± 16%; India: 29 ± 17%; *P* = 0.65), *Lachnospiraceae* (DRC: 21 ± 9%; India 20 ± 8%; *P* = 0.37), and *Ruminococcaceae* (DRC: 18 ± 8%; India: 19 ± 10%; *P* = 0.71), none of which differed significantly between DRC and India. In contrast, *Bifidobacteriaceae* (phylum Actinobacteria) was significantly higher in India (5.5 ± 6.5%) than in DRC (0.4 ± 0.8%; *P* = 1.91E-27). At the genus level, *Prevotella* remained the only strain that was over 10% abundance (DRC: 27 ± 11%; India: 29 ± 13%; *P* = 0.32). Some fiber fermenting strains *Succinivibrio* (DRC: 3.2 ± 0.8%; India: 3.2 ± 0.6%; *P* = 0.11) and *Roseburia* (DRC: 2.0 ± 0.3%; India: 2.1 ± 0.4%; *P* = 0.31) were at relatively high abundance without differences between DRC and India. Consistent with findings of *Bifidobacteriaceae*, the genus *Bifidobacterium* was higher in India (4.95 ± 1.0%) than in DRC (0.3 ± 0.1%; *P* = 2.71E-27), as were *Lactobacillus* (India: 1.2 ± 0.1%; DRC: 0.2 ± 0.0%; *P* = 2.39E-13) and *Faecalibacterium* (India: 8.4 ± 2.9%; *P* = 6.51E-7; DRC: 6.0 ± 1.7%). In contrast, *Ruminococcus* was higher in DRC (2.3 ± 0.7%) than in India (1.8 ± 0.4%; *P* = 3.24E-5). The *Bacteroides*/*Prevotella* ratio was 0.11 ± 0.50 and 0.23 ± 0.90 for DRC and India, respectively (*P* = 0.22).

The only demographical variable that was significantly associated with the gut microbiota composition was the categorical variable “open sewage.” This question asked the participants if they had open sewage near their house. All of the participants in DRC answered “No” while ∼50% in India answered “No” and ∼50% answered “Yes” (Table 1). The genus *Escherichia-Shigella* did not differ by site (DRC vs. India; *P* = 0.39), but was higher in those who reported “No” nearby open sewage compared to those that reported “Yes” in India (2.2 ± 0.8 vs. 0.6 ± 0.7%; *P* = 2.0E-5).

### Associations of Dietary Intakes and the Gut Microbiota

**FIGURE 4:**
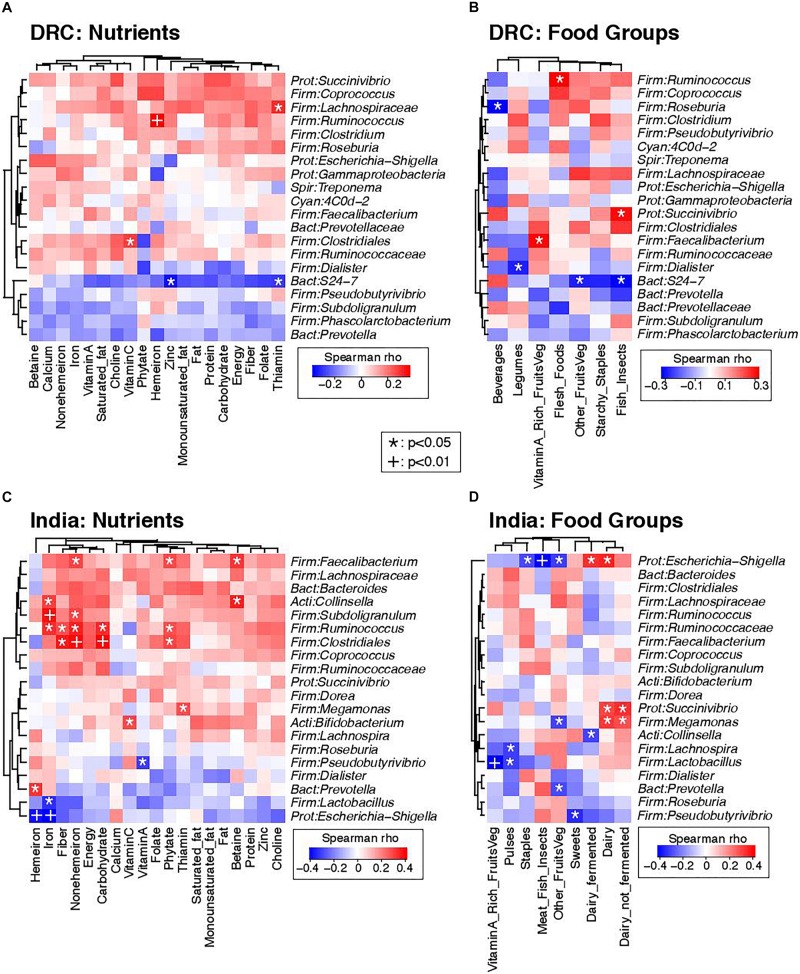
Correlations between dietary intakes and the gut microbiota. Heatmaps summarize Spearman correlation coefficients (rho values) and *p*-values for pairwise comparisons of dietary intakes and bacterial genera. **(A)** Correlations of nutrients and bacterial genera in DRC. **(B)** Correlations of food groups and bacterial genera in DRC. **(C)** Correlations of nutrients and bacterial genera in India. **(D)** Correlations of food groups and bacterial genera in India. To simplify visualization, only the 20 most abundant genus-level taxa are presented in these plots. Nominally statistically significant relationships are indicated by overlying symbols: ^*^p < 0.05; ^+^*p* < 0.01. Dendrograms on the left and top axes of each plot show the results of hierarchical clustering of taxa and nutrients, respectively, based on Euclidean distances.

Strong associations were noted between several dietary intake measures and the gut microbiota. Figure 4 presents heat maps summarizing pairwise Spearman correlation test results between nutrients, food intakes, and the 20 most abundant genera; DRC and India were analyzed separately in order to determine diet-microbiome relationships that were not confounded by country. Figures 4A,C are heat maps of nutrients and the gut microbiota for DRC and India, respectively. *Ruminococcus* was positively associated with iron intake at both sites and with fiber intake in India. The potentially pathogenic genus *Escherichia-Shigella* was negatively associated with iron intake in India. Figures 4B,D are heat maps of food groups and the gut microbiota for DRC and India, respectively. *Succinivibrio* was positively associated with dairy intake in India and fish/insects in DRC (*P* < 0.05). In DRC, *Ruminococcus* was positively associated with flesh foods (*P* < 0.05) and *Faecalibacterium* was positively associated with vitamin A-rich fruits and vegetables. In India, *Escherichia-Shigella* was also negatively associated meat/fish intakes (*P* < 0.01) and positively associated with fermented dairy intakes (*P* < 0.01).

## Discussion

Although the areas of our study in both DRC and India were both rural and low resourced regions, they had distinctive dietary patterns and geographical features. Various studies have shown that diet and geographical location are the two strongest determinants of the gut microbial community structure (O’Keefe et al., 2007; Ou et al., 2013; Vangay et al., 2018). One study showed that host location had the strongest associations with microbiota variations and microbiota-based metabolic disease models developed in one location failed when used elsewhere (He et al., 2018). A recent study (Vangay et al., 2018) showed that United States immigrants from a non-Western country had immediate loss of gut microbial diversity and quickly adopted a gut microbial profile of United States associated strains. These effects increased with the duration of United States residence and were compounded by obesity and across generations (Vangay et al., 2018). Diet and geographical location are usually overlapping and it is challenging to distinguish the effects between the two. One study compared the gut microbiota of Pygmy hunter-gatherers, Bantu farming populations, and Bantu fishing populations in the Southwest Cameroon area (Morton et al., 2015). Although within close proximity, these three cohorts had distinctive gut microbial communities, which differentiated hunter-gatherers from neighboring farming and fishing populations. In the present study, participants in DRC consumed a fair amount of animal protein from caterpillars, termites, and small-fish with minimal dairy intakes, while participants in India were primarily lacto-vegetarian and consumed fermented and non-fermented dairy products on a regular basis. These differences in dietary patterns may have especially contributed to the differences in the gut microbial communities between DRC and India.

Both *Bifidobacterium* and *Lactobacillus* are commonly used probiotics and were enriched in participants from India. *Bifidobacterium* is significantly higher in infants than in adults (Khonsari et al., 2016) due to the digestion of human milk oligosaccharides by some *Bifidobacteria* species (Marcobal and Sonnenburg, 2012). *Bifidobacterium* has previously been associated with dairy food intakes and was found to be higher in United States populations than in agriculturalist communities in Peru (Obregon-Tito et al., 2015), as well as Italians vs. Hadza hunter-gatherers (Schnorr et al., 2014). Indeed, the lower relative abundance of *Bifidobacterium* (0.3%) in DRC was consistent with the fishing, farming and hunter-gatherer populations in South Cameroon (0.1–0.5%) who also have little or no access to dairy products (Morton et al., 2015). Likewise, Indian participants had *Bifidobacterium* at 4.95%, consistent with the United States population (∼4%) (Obregon-Tito et al., 2015). However, we did not find a significant statistical association of dairy (fermented or non-fermented) intake and *Bifidobacterium* in the Indian participants. This may be due to the relatively large variation of dairy food intakes reported, or the lag between gathering the microbiota samples and obtaining the diet records. However, there was also a positive association of fermented dairy intake and potential pathogens of the *Escherichia-Shigella* genus in India. Because these fermented dairy foods (e.g., curd) were typically homemade in an environment of relatively poor hygiene (e.g., with milk cows in proximity to the family home), it is plausible that the home-made fermented dairy was contaminated with *Escherichia-Shigella*.

The bacterial families with the greatest relative abundances in both DRC and Indian participants were commensals *Lachnospiraceae* (20%), *Prevotellaceae* (29%), and *Ruminococcaceae* (18%). These findings were consistent with previous research in Hadza hunter-gatherers of Tanzania (Schnorr et al., 2014), for whom *Lachnospiraceae* (10%), *Prevotellaceae* (6%), and *Ruminococcaceae* (34%) were also the three most abundant families. All three families include member species that can degrade complex polysaccharides and are considered potent short-chain fatty acid producers. The lack of direct association between fiber intake and these bacterial taxa may be due to the lag between collections of microbiota samples and diet records, which may have weakened some of the correlations between microbiota and dietary data, especially if diets changed in early pregnancy. Also, dietary data from two 1-day recalls may not be sensitive enough to link with individuals’ biomarkers, including the microbiota, although the monotony of the typical diets in these populations tends to mitigate that concern. Finally, because dietary data were available from only one-half of the participants in each site for this analysis, statistical power was reduced. However, the overall dietary patterns observed from the diet records still were consistent with agricultural communities. At the genus level, genera *Ruminococcus* has frequently been reported to be depleted in agriculturalist communities compared with urban communities (Schnorr et al., 2014; Obregon-Tito et al., 2015) and the abundance of this genus may be diet-dependent. In the present study, *Ruminococcus* was higher in DRC than in India and was positively associated with intake of animal-flesh foods. This was consistent with previous reports that the abundance of *Ruminococcus* increased with meat intake in infants (Laursen et al., 2016) and animal models (Zhu et al., 2015). Species within *Ruminococcus* are butyrate producers, and undigested proteins and amino acids in the colon may serve as an additional substrate for short-chain fatty acid production along with non-digestible carbohydrates (Rasmussen et al., 1988). Similar to *Ruminococcus*, *Succinivibrio*, another possible short-chain fatty acid producer that has been associated with high fiber diets (Graf et al., 2015) was also positively associated with protein intake (i.e., with dairy intake in India and fish/insects in DRC), suggesting the potential role of protein in the modulation of gut microbiota and its metabolites (Graf et al., 2015). Another interesting finding was that the abundance of potential pathogen *Escherichia-Shigella* was higher in those who reported not having open sewage. The participants who answered “no” in fact did not have any sewage system and thus more likely to have open defecation, with potentially greater exposure to potential pathogens.

Consistent with previous research, we found that the family *Prevotellaceae* and its genus *Provetella* were highly abundant in both DRC and Indian participants (∼27%). *Prevotella* is strongly associated with plants and fiber intakes and can be used to differentiate agriculturalist and urban communities (Wu et al., 2011). Most urban communities have a relatively low *Provetella* abundance of less than 5% while it typically is one of the most abundant family/genera in agriculturalist communities (De Filippo et al., 2010). Bacteroidetes have two main genera, *Bacteroides* and *Prevotella*, and multiple observations have shown a reciprocal relationship, such that individuals with high abundance of *Prevotella* usually have lower levels of *Bacteroides* (O’Keefe et al., 2007; Ou et al., 2013). In the present study, the abundance of *Bacteroides* (<0.001%) was low in both DRC and India, and the *Bacteroides/Prevotella* ratio was also low. A recent study (Kovatcheva-Datchary et al., 2015) demonstrated that a 3-day barley kernel diet intervention improved glucose metabolism only in individuals with an increase of *Prevotella copri* and higher *Prevotella/Bacteroides* ratio. Overall, these findings suggest substantial dietary influences on the structure of the gut microbiota in individuals from two distinctive geographical locations but having certain similarities between their dietary patterns (high in fiber and plant-based foods).

The richness of the gut microbiota (i.e., the number of bacterial taxa present in a given specimen) was higher in DRC than in India. Emerging research has shown that diet diversity is positively associated with richness within gut microbiota (Heiman and Greenway, 2016). In the present study, more than 60% of participants in India had adequate diet diversity, based on the consumption of ≥5 primary food groups (Food and Agricultural Organization of the United Nations, 2016), while less than 30% of DRC women met this criterion. Several studies have found that omnivorous individuals have higher bacterial richness compared to vegetarians (Zimmer et al., 2012; Ferrocino et al., 2015) and this may partially explain the higher bacterial richness in DRC (primarily omnivorous with regular consumptions of animal-based foods) compared with India (lacto-vegetarian).

To our knowledge, this is the first study to directly compare gut microbial composition and diversity in women of child-bearing age in DRC and India, and to link these with quantitative dietary intakes. Strengths of this study include a relatively large sample size, using repeat 24-h dietary recalls and addressing understudied populations. There are also several limitations. First, the 24-h dietary recall data were available for only a subgroup of participants and were obtained at the end of the first trimester of pregnancy, which may not reflect their habitual dietary intakes. Although mid-late pregnancy is associated with changes in the gut microbiota (Koren et al., 2012), the fecal samples for the participants in this study were all obtained prior to conception. In our settings in DRC and India, however, diets are quite monotonous and are unlikely to change substantially with pregnancy. We recognize the challenges of accurately measuring dietary intakes, but data presented are also likely among the most carefully obtained dietary data for pregnant women and women of reproductive age in these settings. Second, only women were included in this study due to the nature of the WF intervention, and findings cannot be simply extrapolated to men. Third, emerging research also showed different gut microbiota in a population with varied ethnic origins but shared geography, independent of diet (Deschasaux et al., 2018). Because DRC and India also have distinctive ethnicity, it is challenging to differentiate the effect of diet from ethnicity.

In conclusion, diet is an influential determinant of the gut microbiota of women in DRC and India. The relationship between diet and gut microbiota was much stronger than demographic factors such as socioeconomic status. Consumption of plant-based foods, animal-flesh foods, and fermented dairy foods all showed evidence of associations with gut microbiota.

## Data Availability

The datasets generated for this study can be found in the NCBI https://www.ncbi.nlm.nih.gov/sra/?term=PRJNA553183.

## Ethics Statement

This study was approved by the Institutional Review Board at each participating site and registered at ClinicalTrials.gov (NCT01883193).

## Author Contributions

KH and NK conceived the original idea and designed the initial protocol. AT, AL, SG, SD, JW, and MS conducted the procedures at DRC and India sites. DF, DI, CR, and JK analyzed the stool samples and processed the microbiota data. RL analyzed the dietary data. MT and DF performed the statistical analysis. MT wrote the report with close collaboration with NK, DF, and AH, and with input from all authors. All authors read and approved the final version of the manuscript.

## Conflict of Interest Statement

The authors declare that the research was conducted in the absence of any commercial or financial relationships that could be construed as a potential conflict of interest.
